# Socket Instruments

**Published:** 1880-11

**Authors:** 


*Socket Instruments* are old in idea, and the itinerant dentist of years past, whose "kit" was purposely made as light as possible, devised various handles for holding bits, more or less clumsy and liable to motion in the joint. Neat, light handles for excavators and pluggers, (cannot we get rid of that ugly word "plugger?") are soon spoiled in our attempts at repointing, and a handle into which the point can be easily screwed and removed, and which can be kept neat and bright, is a desideratum. This the Messrs. Johnston Bros., of New York, have done some time since, supplying the profession with a set of light steel nickel plated handles, beautifully milled in various shapes, for convenience of recognition, and from which the points of excavators &c. can be readily removed, and in which they are held firmly. As combining beauty and utility these are the nearest to perfection of any instruments we have seen. H.

